# Minimizing contrast agent dosage in CT angiography using a saline chaser with a low trigger threshold

**DOI:** 10.3389/fradi.2025.1709609

**Published:** 2026-01-26

**Authors:** Linghan Li, Xiaojian Zhu, Xunhua Wu

**Affiliations:** 1 Department of Radiology, Changsha Hospital of Traditional Chinese Medicine (Changsha Eighth Hospital), Changsha, China; 2 Department of Radiology, Chaling People’s Hospital, Zhuzhou, Hunan, China; 3 Department of Radiology, Xiangya Hospital, Central South University, Changsha, Hunan, China

**Keywords:** computed tomography angiography, contrast-induced nephropathy, image quality, iodine contrast agent, saline chaser

## Abstract

**Background:**

Computed tomography angiography (CTA), utilizing iodinated contrast agents, is a first-line diagnostic tool for cardiovascular diseases (CVD). However, the administration of contrast agents introduces potential risks to patients.

**Objective:**

This study aims to design a novel protocol for CTA that integrates a saline chaser strategy and low-threshold triggering and evaluate, and demonstrate its feasibility and practicality minimizing the required contrast agent for imaging.

**Methods:**

A novel protocol for CTA incorporating a saline chaser strategy and low-threshold triggering was developed. Random assignment of eighty patients undergoing CTA examinations divided them into conventional and novel protocol groups. The assessment encompassed iodine intake, radiation dose, image quality, and superior vena cava artefacts in both cohorts.

**Results:**

The novel protocol group exhibited a noteworthy 20% reduction in iodine intake compared to the conventional group (*P* < 0.05, FDR correction). Notably, Hounsfield units (HU) of the ascending and descending aorta at the T12 level tended to be lower in the novel protocol group (*P* < 0.05, uncorrected), while parameters like signal-to-noise ratio (SNR) and contrast-to-noise ratio (CNR) exhibited no significant between-group differences. Compared with conventional group, fewer superior vena cava artefacts were observed in the novel protocol group, and subjective image quality assessment by physicians remained consistent between the two groups (kappa = 0.84, *P* < 0.01).

**Conclusion:**

The combination of saline chaser strategy with low-threshold triggering in CTA imaging proves a viable approach, significantly curtailing the utilization of iodinated contrast agents, and superior vena cava artefacts.

## 1 Introduction

Cardiovascular disease (CVD), chiefly characterized by ischemic heart disease and arterial conditions such as stroke, is a significant contributor for global mortality and disability rates (1, 2). Computed tomography angiography (CTA) is a frontline diagnostic tool for assessing arterial health, encompassing both the aorta and coronary arteries (3). Enhancing vascular imaging through CTA necessitates using iodinated contrast agents (4). However, employing these agents poses potential health risks, including Contrast-Associated Acute Kidney Injury (CA-AKI), recognized as an inducer of Acute Kidney Injury (AKI) (5, 6). Notably, CA-AKI accounts for 11% of hospital-related AKI cases, ranking third among causes of iatrogenic kidney injury (7, 8). Consequently, reducing contrast agent dosage and augmenting CTA safety via multifaceted strategies are central tenets of clinical research.

CTA employs various techniques to minimize patient iodine exposure while upholding image quality (9, 10). One such technique involves the saline chaser method, which facilitates the uniform distribution of contrast agents within blood vessels, optimizing vascular opacification (11–14). Studies underscore the use of the physiologic saline solution as an optimal supplementary injection, delivered at a rate of 4–5 mL per second, yielding maximal attenuation in the aorta or coronary arteries (15). Another strategic approach involves adjusting CTA trigger thresholds. Multiple studies propose trigger thresholds for aortic CTA protocols within the 150–350 Hounsfield Units (HU) range (16). Innovative research explores real-time monitoring techniques, dynamically adjusting trigger thresholds based on variations in contrast agent concentration within regions of interest (17). This precision tuning mechanism ensures accurate threshold determination, effectively reducing contrast agent dosages while preserving image quality. However, existing clinical research has yet to integrate multiple strategies, and current equipment struggles to integrate the methods mentioned earlier effectively.

Hence, we hypothesize that the synergistic application of a saline chaser strategy and low-threshold triggering can significantly diminish patient iodine exposure without compromising image fidelity. Based on this hypothesis, we developed a novel device termed the “Low-Threshold Trigger with Saline Chaser” and test its function and feasibility in an initial clinical trial. This study aims to comprehensively assess the device's function from a series of indicators including clinical feasibility, iodine intake, radiation dosage, superior vena cava artefact, and vascular image quality.

## 2 Materials and methods

### 2.1 Protocol design

**Figure 1:**
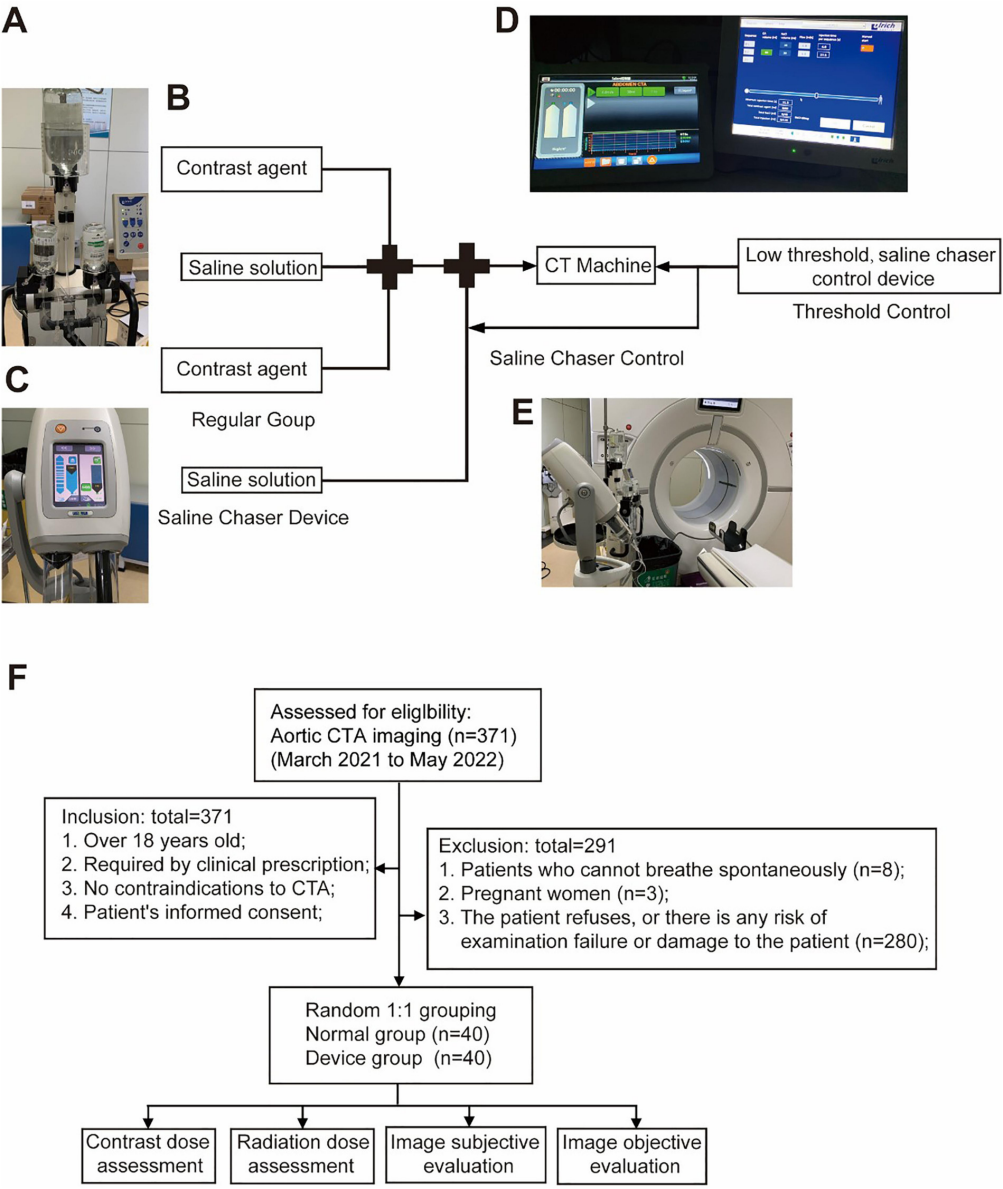
Schematic and photographic representation of the device. **(A)** Overall schematic representation of the novel protocol scanning, comprising four main components. **(B)** Injector for administering regular contrast agent. **(C)** The saline chaser is regulated by a control system and administered via a three-way valve. **(D)** Low threshold and saline chaser control device. **(E)** CT scanning device. **(F)** The flow chart illustrates the sequence of the study process. Following the inclusion and exclusion process, 80 patients were randomly assigned to two groups in a 1:1 ratio: the conventional group and the novel protocol group. Subsequently, these groups underwent assessments for contrast agent dosage, radiation dosage, and subjective and objective image evaluations.

The schematic diagram of the novel protocol scanning design and its physical representation is shown in Figure 1. The apparatus comprises four components (Figure 1A). The standard iodinated contrast agent injection unit is the regular contrast agent injection device (Figure 1B). The saline chaser device (Figure 1C) is equipped with a three-way valve, connecting one outlet to the patient, while an additional saline chaser injector, exclusive of the contrast agent injection mode, administers saline solely under the control of the control unit (Figure 1D). Concurrently, the control unit regulates the trigger threshold of the CT machine (Figure 1E). The novel protocol aims to achieve CTA with diminished contrast agent application, upholding image quality. This goal is attained through a straightforward and practical design, incorporating the saline chaser and a low trigger threshold of CTA.

### 2.2 Experimental design

This study received approval from the Ethics Committee of Xiangya Hospital (Approval No. 202012237), and informed consent was obtained from all enrolled patients from March 2021 to May 2022. The inclusion criteria encompassed the following: (1) age over 18 years; (2) clinical prescription for CTA; (3) absence of contraindications to CTA; and (4) patient's informed consent. Exclusion criteria encompassed (1) patients unable to breathe spontaneously, (2) pregnant women, and (3) patients who declined participation or were susceptible to examination failure or harm. The study flowchart is presented in Figure 1F.

After enrollment, patients were assigned to either the conventional group or the novel protocol group based on a computer-generated random number list (specifically, using the RAND function in Excel) in a 1:1 ratio.

### 2.3 Scan protocol and image post-processing

The CTA scans were conducted utilizing a 256-slice Revolution CT scanner manufactured by GE Healthcare. The scan encompassed a range from 5 cm above the apex of the lungs down to the level of the pubic symphysis. The layer thickness and interval parameters were configured at 0.625 mm, and the collimator was finely tuned to 128 mm × 0.625 mm. For contrast enhancement, both study groups were administered injections of a non-ionic contrast agent (350 mg/mL iohexol) via a high-pressure syringe (German Ulrich Missouri XD2001). The syringe was equipped with a 20 G indwelling needle and was inserted into the antecubital vein.

The conventional group scan introduced the contrast agent through the cubital vein. The contrast agent dosage was calculated based on the individual's body weight, with a factor of 1.5 mL per kilogram, and was administered at a flow rate of 4.0 mL/s. The central horizontal aorta at the T8 level was designated as the region of interest (ROI), employing a threshold of 180 CT number, Hounsfield Unit (HU). The image acquisition occurred approximately 5.9 s after attaining the contrast agent's peak concentration.

In the novel protocol group scan, the initial high-pressure injector administered the contrast agent into the cubital vein at a velocity of 4.0 mL/s. The ROI for the central aorta at the T8 level was configured to 60 HU. Upon reaching the peak value, the first injector was halted, and the second high-pressure injector was engaged to infuse 30 mL of normal saline. When the T8 level is observed to trigger the scan, the injection of the contrast agent is stopped, and the saline chaser is started at the same time. The initiation of scanning was delayed by 8 s.

After acquiring thin-slice data for aortic image reconstruction, the data were transmitted to the AW4.7 workstation. The aortic analysis was performed following processing using the software suite integrated with the GE CT scanner. The 3D post-processing encompassed multiplanar reconstruction (MPR), curved planar reconstruction (CPR), maximal intensity projection (MIP), and volume rendering (VR) techniques.

The detailed scanning precedures for different clinical indications could be found in the Supplementary Material.

### 2.4 Contrast agent dosage and radiation dose assessment

The quantification of contrast agents was articulated using iodine intake as the metric, with iodine intake (g) calculated as the product of iodine contrast agent volume (mL) and contrast agent concentration divided by 1,000. Radiation dosage parameters encompassed the volume CT dose index (CTDIvol, mGy), CT dose-length product (DLP, mGy·cm), and effective radiation dose (ED, mSv), the latter expressed as the product of DLP and a conversion factor denoted as “*k*”, where *k* represents the radiation weight conversion factor. The “*k*” value is 0.017 mSv/(mGy·cm).

### 2.5 Objective evaluation of image quality

The quantitative image quality assessment encompasses specific HUs, signal-to-noise ratio (SNR), and contrast-to-noise ratio (CNR). HU was determined for various anatomical points: the ascending aorta, descending aorta at the T12 level, residual imaging of the superior vena cava, and the left external iliac artery. A region of interest (ROI) measuring 5 mm² was applied. Triplicate measurements were conducted at each designated point, with the standard deviation (SD) of HU taken from the right erector spinae muscle at the same level serving as the background noise reference. ROIs were meticulously positioned to exclude regions with uneven density. The mean value was computed for each corresponding measurement. The aortic SNR was computed using the subsequent formula:

$$\mathrm{SNR}=\frac{\mathrm{Aortic}}{\mathrm{Noise}\;}$$

And CNR (contrast signal-to-noise ratio):

$$\mathrm{CNR}=\frac{\left(\mathrm{Aortic}\text{-}\mathrm{Erector}\;\mathrm{spinae}\right)}{\mathrm{Noise}}$$

### 2.6 Subjective evaluation of image quality

Two experienced radiologists (LL and LK) employed a double-blind approach to assess aortic and superior vena cava images. They used a subjective 4-point scale to evaluate image quality based on the following criteria:

- 4 points: Excellent image quality, featuring clear 3D reconstruction images, smooth and sharply defined blood vessel edges, and no cross-layer artefacts.
- 3 points: Relatively clear image quality, displaying well-defined blood vessel edges, minimal misalignment artefacts, enabling accurate diagnosis.
- 2 points: Average image quality, characterized by 3D reconstruction artefacts, requiring diagnosis through tomographic combination.
- 1 point: Poor image quality, with substantial artefacts hindering diagnosis.

Scores of 3 and 4 points on the subjective image quality assessment indicated satisfactory diagnostic criteria.

For the assessment of superior vena cava image quality, a 3-point scale was used, focusing primarily on artefact presence within the vein:

- 3 points: No artefacts within the veins.
- 2 points: Few artefacts within the vein, not obstructing observation of peripheral arteries and blood vessels.
- 1 point: Numerous artefacts significantly affecting peripheral artery observation.

### 2.7 Data analysis

Statistical analysis was conducted using IBM SPSS Statistics for Windows, Version 22.0 (IBM Corp., Armonk, NY, USA). The measurement data were expressed as $\left(\bar{X}\pm s\right)$, and the *t*-test was employed for inter-group comparison analysis. The count data were presented as frequencies, and the *χ*² test was used to assess the between-group differences. The agreement between subjective scores was evaluated using Cohen's kappa test, and the kappa values were interpreted as follows: poor agreement (0.00–0.20), fair agreement (0.21–0.40), moderate agreement (0.41–0.60), substantial agreement (0.61–0.80), and almost perfect agreement (0.80–1.00). Statistical significance was set at *P* < 0.05. To address the issue of multiple comparisons, the False Discovery Rate (FDR) was controlled using the Benjamini-Hochberg (BH) procedure.

## 3 Results

### 3.1 Patient groups and comparability

The two patient groups demonstrated strong comparability. All 80 included patients successfully underwent CTA examinations and exhibited favourable procedural tolerance. The conventional group consisted of 33 males and 7 females, with an average age of 57.4 ± 13.2 years and an average body mass index (BMI) of 23.1 ± 3.0 kg/m^2^. Meanwhile, the novel protocol group comprised 32 males and 8 females, with an average age of 57.3 ± 12.6 years and a BMI of 23.3 ± 4.3 kg/m^2^. Notably, there were no between-group significant differences in sex, age, age group, height, weight, or BMI, indicating robust comparability (Table 1).

**Table 1:** Clinical characteristics.

Characteristics	Conventional group (*N* = 40)	Novel protocol group (*N* = 40)	*P*-value
Sex			
Female	7	8	0.500
Male	33	32	
Age	57.4 ± 13.2	57.3 ± 12.6	0.847
<55	18	16	0.411
≥55	22	24	
Height (cm)	167.03 ± 7.15	167.55 ± 9.43	0.780
Weight (kg)	64.89 ± 11.24	64.87 ± 9.98	0.996
BMI (kg/m^2^)	23.31 ± 4.23	23.10 ± 3.01	0.608
Region of interest for test			0.324
Abdominal aorta	4	3	
Coronary artery	5	8	
Iliac artery	1	0	
Heart valve	3	8	
Aorta	27	21	
Surgery condition			0.410
Post-surgery	15	17	
Preoperative or evaluation	25	23	

Value is reported as mean ± standard deviation.

### 3.2 Clinical indications and key test points

Furthermore, we obtained clinical indications for examination and identified key observation pionts (Table 1). Importantly, no significant differences were observed between the two groups (*P* = 0.324). The detailed data for each test point is displayed as follows: the abdominal aorta (4 vs. 3), coronary artery (5 vs. 8), iliac artery (1 vs. 0), heart valve (3 vs. 8), and aorta (27 vs. 21). Interestingly, our analysis revealed that over fifty per cent of patients in both groups had previously undergone CTA assessments and even interventional procedures (25 vs. 23, *P* = 0.410).

### 3.3 Quantitative and semi-quantitative assessment of image quality

Quantitative image quality did not exhibit any discernible differences between the two groups; however, the device group displayed fewer artefacts in the vena cava. Key indicators for objective assessment encompass HU, signal-to-noise ratio (SNR), and contrast-to-noise ratio (CNR) for specific anatomical regions, including the ascending aorta, descending aorta, left external iliac artery, and superior vena cava at the T12 level (Table 2). No sifnificant differences were observed in the comparison of HU for the left external iliac artery and superior vena cava. Subjects in the patients in conventional group showed a tendency of higher HU values than that in the novel protocol group for the ascending aorta (462.17 ± 36.78 vs. 446.54 ± 32.95, *P* = 0.049, *P*_FDR corr._ = 0.245) and descending aorta at the T12 level (458.62 ± 36.35 vs. 443.06 ± 28.44, *P* = 0.036, *P*_FDR corr._ = 0.245). SNR and CNR results indicated slightly superior measurements in the conventional group for the ascending aorta, descending aorta at the T12 level, and left external iliac artery. However, these differences lacked statistical significance (*P* > 0.05).

**Table 2:** Comparison of objective scores of image quality between the two groups $\left(\bar{X}\pm s\right)$.

Measurement items		Conventional group	Novel protocol group	*t-*value	*P* value	*P*_FDR corr._
HU	AA	462.17 ± 36.78	446.54 ± 32.95	2.002	**0.049**	**0.245**
	DA	458.62 ± 36.35	443.06 ± 28.44	2.132	**0.036**	**0.245**
	EIA_L	455.01 ± 35.75	428.96 ± 43.69	1.235	0.208	0.393
	SVC	161.30 ± 31.82	160.77 ± 20.52	0.088	0.170	0.393
Image noise						
SNR	AA	17.34 ± 2.77	17.16 ± 3.09	0.271	0.787	0.841
	DA	15.16 ± 2.84	14.62 ± 2.76	0.862	0.391	0.489
	EIA_L	15.07 ± 2.33	14.50 ± 1.72	1.253	0.214	0.393
CNR	AA	15.27 ± 2.54	15.15 ± 2.90	0.201	0.841	0.841
	DA	13.17 ± 2.69	12.60 ± 2.50	0.988	0.326	0.466
	EIA_L	13.23 ± 2.21	12.70 ± 1.75	1.194	0.236	0.393

Value are reported as mean ± standard deviation; SNR, signal noise ratio; CNR, contrast-to-noise ratio; AA, ascending aorta; DA, descending aorta; EIA-L, left external iliac artery; SVC, superior vena cava.

Bold values indicate statistically significant differences or corrected *P*-values.

**Figure 2:**
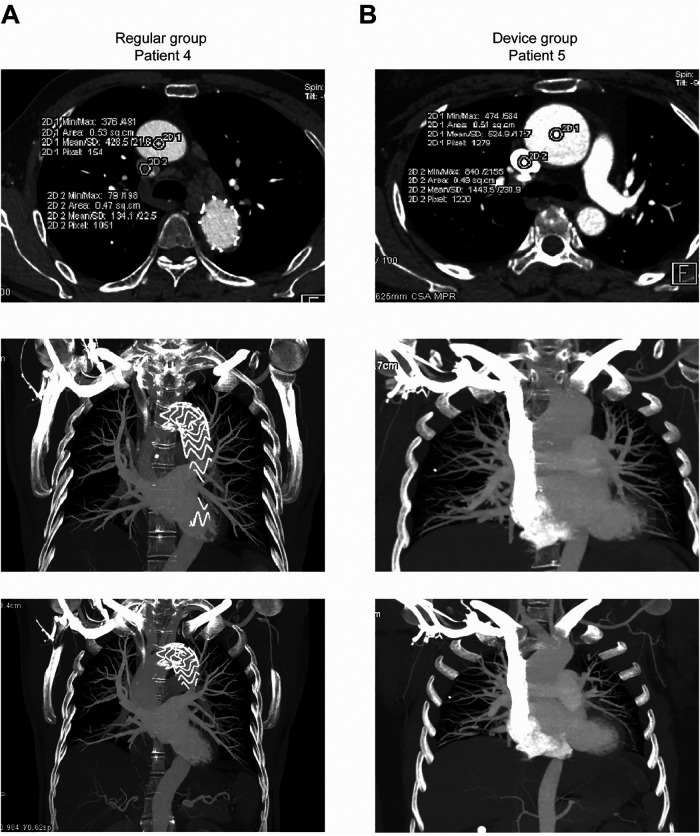
Comparative analysis of device group and regular group images. **(A)** Axial and maximum intensity projection views of the superior vena cava in the conventional group were obtained following aortic stent implantation in Patient 4. Noticeable pseudofiltering artefacts were observed in the superior vena cava images. **(B)** Patient 5, included in the novel protocol group, underwent computed tomography pulmonary angiography (CTPA). The image depicts CTPA findings specific to patients within the novel protocol group. Enhanced imaging of the superior vena cava is evident.

**Figure 3:**
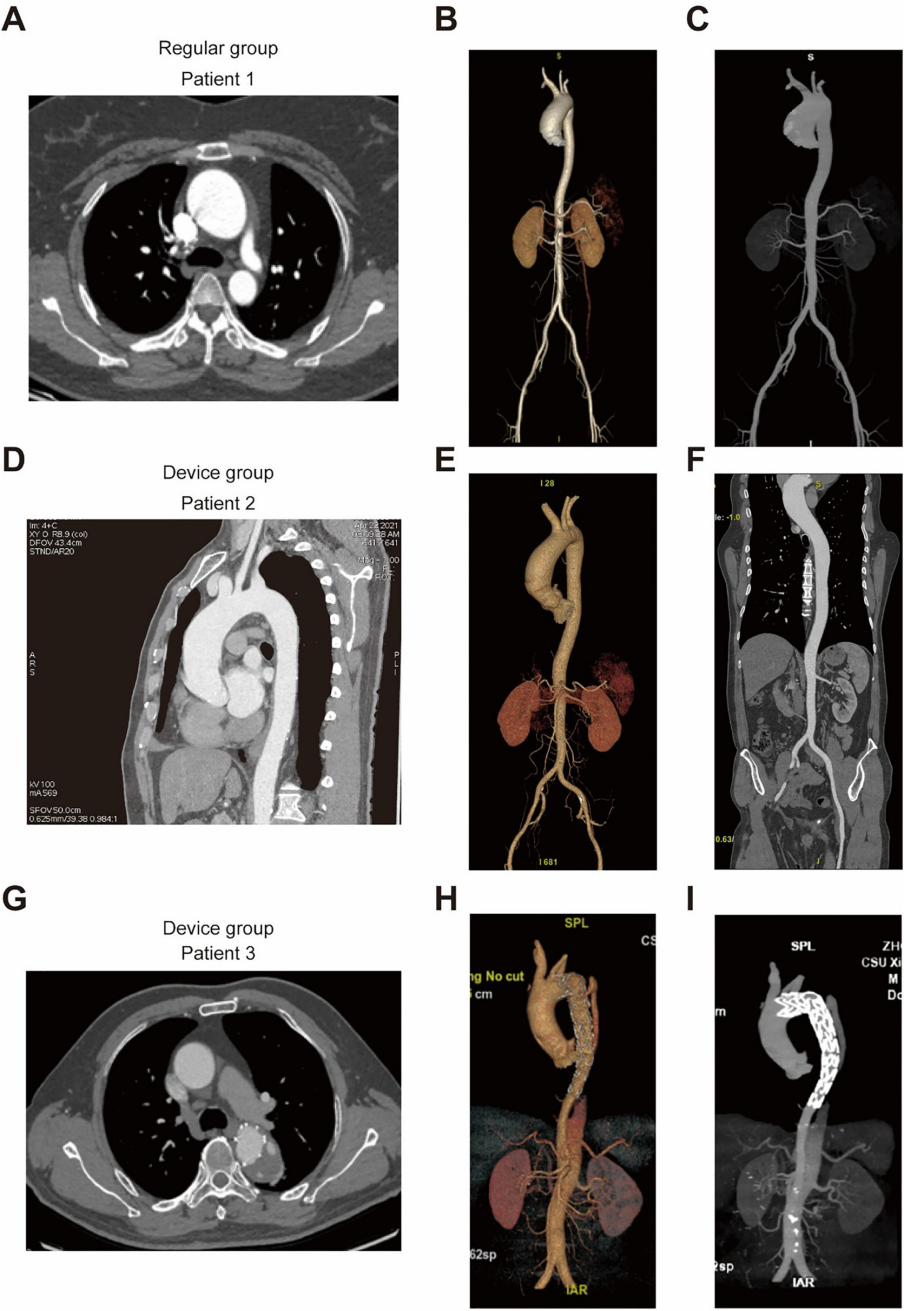
Improved image quality of the superior vena Cava in the device group. **(A–C)** Patient 1, representative of the conventional group, underwent imaging with 80 mL of contrast agents. **(A)** Axial view of the superior vena cava, **(B)** Aortic Volume Rendering (VR), and **(C)** Maximum Intensity Projection (MIP) demonstrate well-developed contrast enhancement. The images are sharp and clear; however, a substantial amount of contrast agent within the superior vena cava interferes with the visualization of the aorta. **(D–F)** Patient 2, belonging to the novel protocol group, received 60 mL of contrast agents. **(D)** Sagittal view of the superior vena cava, **(E)** Aortic VR, and **(F)** Coronal view exhibit excellent image quality with distinct clarity. **(G–I)** Patient 3, also from the novel protocol group, was administered 60 mL of contrast agents post-aortic stent implantation. **(G)** Axial view of the superior vena cava, **(H)** Aortic VR, and **(I)** MIP demonstrate high-quality imaging devoid of artefacts while depicting an unobstructed superior vena cava.

Semi-quantitative assessment of arterial images displayed substantial agreement between the two groups (kappa = 0.844, *P* < 0.001), as outlined in Table 3 and Figure 2. Compared with the conventional group, subjects in the novel protocolgroup showed fewer artefacts in superior vena cava, indicative of superior image quality (Table 4 and Figures 2, 3), and our subgroup analyses further supports these results (See Supplementary Tables S1–S4, Supplementary Figures S1–S4). Additionally, interventional procedures displayed no substantial disparity between the groups (25 vs. 23, *P* = 0.410).

**Table 3:** A subjective index score of aortic image quality.

Group	Cases	Image quality subjective index score				Kappa	*P*-value
		4	3	2	1		
Conventional group	40	32	6	1	1	0.844	**<0** **.** **001**
Novel protocol group	40	33	5	2	0		

Bold values indicate statistically significant differences or corrected *P*-values.

**Table 4:** A subjective index score of superior vena cava image quality.

Group	Cases	Image quality subjective index score			Kappa	*P*-value
		3	2	1		
Conventional group	40	33	5	2	0.167	0.134
Novel protocol group	40	38	2	0		

### 3.4 Intergroup analysis of iodine intake and radiation dose

The iodine intake in the conventional group (26.02 ± 1.23 g) was significantly lower than that in the device group (20.81 ± 0.95 g) (*P* = 0.001), and our subgroup analyses further supports these results (see Supplementary Tables S1–S4, Supplementary Figures S1–S4). Patients in the conventional group received a slightly higher CTDIvol (mGy) value than those in the novel protocol group (8.37 ± 0.56 vs. 8.01 ± 0.76, *P* = 0.017). It is important to note that despite the reduction in iodine load in the device group, no significant intergroup differences of DLP and ED were observed (*P* > 0.05), as shown in Table 5.

**Table 5:** Comparison of radiation dose and iodine load between the two groups of images $\left(\bar{X}\pm s\right)$.

Measurement items	Conventional group	Novel protocol group	*P*-value
Contrast agent dosage (mL)	74.25 ± 3.50	59.50 ± 2.73	**<0.001**
Iodine intake (g)	26.02 ± 1.23	20.81 ± 0.95	**<0.001**
CTDI_vol_ (mGy)	8.37 ± 0.56	8.01 ± 0.76	**0.017**
DLP (mGy·cm)	570.10 ± 57.56	592.12 ± 74.69	0.144
ED (mSv)	9.69 ± 0.98	10.07 ± 1.27	0.144

Value are reported as mean ± standard deviation; CTDIvol, volume CT dose index; DLP, dose-length product; ED, effective dose.

Bold values indicate statistically significant differences or corrected *P*-values.

## 4 Discussion

This is a comprehensive study to use a series clinical and imaging indicators to evaluate the effectiveness and clinical feasibility of our newly developed device for CTA examination. Our quantitative analysis of image quality showed that subjects in the conventional group had a tendency of higher HU values than that in the device group in the ascending aorta and descending aorta at the T12 level. The following semi-quantitative analysis of image quality showed that subjects in the device group showed fewer artefacts in superior vena cava. These findings suggest that the image quality of the device group was at least on par with that of the conventional group. More importantly, our intergroup analysis revealed that subjects in the device group had significantly lower iodine intake than that in the conventional group, indicating that this innovative approach has superiorities in managing iodinated contrast allergies or contrast agents-related biological injury without compromising image quality.

In this study, the final configuration of our apparatus resulted in a reduction of contrast agent dosage by 20% while maintaining imaging quality comparable to the conventional group. Mechanistically, CTDIvol, DLP, and ED are key indicators for assessing CT imaging radiation doses (18). Our findings demonstrate a reduction of about 4% in radiation dose in the equipment group, with no significant differences observed in DLP and ED, indicating consistent radiation conditions during scanning. During the analysis of aortic image quality, aortic HU, SNR, and CNR serve as important objective benchmarks. The results show very small differences in HU, SNR, and CNR, suggesting that our device effectively reduces contrast agent consumption while maintaining image quality following clinical diagnostic standards. Following the low-threshold trigger, contrast agent administration is halted, and 30 mL of saline tracer is administered to ensure sufficient contrast agent volume within the aorta before performing an 8 s delayed scan. Furthermore, increasing the volume of physiological saline injection reduces residual contrast agents within the superior vena cava, minimizing the impact of excessive contrast agents on aortic images and maintaining image quality. We believe that with the application of emerging technologies such as deep learning and personalized contrast agent usage protocols, it is possible to reduce further the dosage of contrast agents and the radiation dose patients receive. This holds the promise of being a progressive approach (19, 20).

Such significant benefits of lower iodine intake, while maintaining comparable image quality, from this newly developed device are crucial for our clinical practice. As is well known, contrast agents-related Acute Kidney Injury (CA-AKI), an important part of iatrogenic renal impairment, is particularly common in CT angiography (CTA) studies (21, 22). It has garnered significant clinical attention due to its high incidence (5%–20%) in patients with risk factors (23). The pathogenesis of CI-AKI is considered to be closely related to the typical hypoxic/toxic injury resulting from changes in renal microcirculation, hypoxia, and reactive oxygen species-mediated cell damage (23–25). The impaired renal function in turn increased susceptibility to contrast agents. In addition to the renal impairment, the over usage of iodine contrast agents can cause damage to the thyroid gland (26). A rencent study with large sample size (more than 4 million patients) found that iodine exposure was linked to an increased risk of thyroid dysfunction (27). In some extreme clinical scenarios, patients may be exposed to significantly higher doses of contrast agents and radiation. For example, once patients undergo an interventional operation, they may experience multiple injections of contrast agents, such as multiple interventional operations, postoperative evaluation, and follow-up re-examinations. To solve these clinical issues, we introduces a novel approach that combines a saline chaser with a low-threshold triggering mechanism. This apparatus reduces contrast agent consumption and radiation exposure without compromising image quality, which are prelimilarily validated in our cohort, significantly improving its clinical utility.

While this study did not demonstrate a significant reduction in per-scan effective dose, the novel protocol presents a potential pathway for cumulative dose reduction in clinical practice. By substantially improving first-pass success rate and image quality (as shown in our results), it may reduce the need for repeat scans due to non-diagnostic images. Furthermore, the faster acquisition could decrease the number of required monitoring phases in complex cases. Future studies focusing on workflow and repeat-scan rates are warranted to quantify this potential benefit.

Our newly developed equipment demonstrates significant advantages even in complicated clinical scenarios. For instance, with the increasing ageing population, various forms of CVD necessitate a substantial demand for clinical applications of CTA (28, 29). In this study, we enrolled a cohort consisted of patients with various conditions including post-implantation diagnosis, screening, and assessment for conditions involving the heart, valves, aorta, abdominal aorta, and pulmonary system. As reported in previous studies, The saline chaser strategy or the approach of reducing the CTA trigger threshold aim to minimize contrast agent usage and contrast agents-associated risks (30–32). Nevertheless, their demerits could not be ignored. For example, Choi's et al. found that the saline chaser strategy might compromise image quality for small vessel imaging, affecting diagnostic accuracy (11). Another imaging technique study reported that lowering the CTA trigger threshold might elevate image noise and decrease clarity (33). Being different from these techniques, the newly designed equipment in our study ingeniously overcomes such tricky clinical issues. The saline chaser we used here offers more precise control on the contrast agent injection rate and pressure, thereby maintaining image quality. Meanwhile, the low-threshold triggering we adopted in this study can reduce patient's exposure to radiation.

Several limitations should be addressed in this study. First, the research relies on a single-centre sample, introducing operator variability. Second, although diverse clinical scenarios are explored, broader applicability requires validation through multicenter, large-sample studies. Third, our study was designed as a pilot comparative analysis; thus, while the sample size was sufficient to detect key differences, it was not formally powered based on a pre-specified effect size, nor did it employ a pre-defined non-inferiority margin. A future trial aimed at formally demonstrating non-inferiority would need to prospectively establish such a clinically meaningful margin and calculate the sample size accordingly. The findings from our present work provide essential preliminary data to guide that process. Four, while patient allocation was performed via computer-generated simple randomization, we acknowledge that formal allocation concealment (e.g., sealed envelopes) was not implemented. Future definitive trials would benefit from incorporating such measures to minimize selection bias. Furthermore, the study possesses untapped potential; there is room for further optimization regarding the relationship between the saline chaser's speed, dose, and trigger threshold. However, recognizing these limitations directs future research to enhance and promote this innovative high-pressure injector and low-threshold triggering strategy.

## 5 Conclusion

Using a saline chaser with a low-threshold trigger for CTA, which involves lower iodine intake without sacrificing image quality, is a viable and promising method for clinical practice.

## Data Availability

The original contributions presented in the study are included in the article/Supplementary Material, further inquiries can be directed to the corresponding authors.
