# Supplementary material for: Minimizing contrast agent dosage in CT angiography using a saline chaser with a low trigger threshold
Source: Front Radiol. 2026 Jan 26;5:1709609. doi: 10.3389/fradi.2025.1709609 (PMC12883799; doi:10.3389/fradi.2025.1709609)
Supplement: Supplementary file 1 [file Datasheet1.pdf]

Supplementary materials and methods

The specific parameters for our novel protocol of Low-Threshold Trigger with Saline Chaser, which were tailored to the clinical indication, are detailed below to facilitate replication:

1. For Aortic CTA:

Trigger Location and Threshold: A circular region of interest (ROI) was placed in the descending aorta at the level of the 8th thoracic vertebra (T8). For patients with prior endovascular stent grafts, the ROI was placed in an aortic segment free of stent material to avoid interference. The automatic triggering threshold was set at 60 HU.

Trigger-to-Scan Delay: Upon reaching the threshold, a standard delay of 8 seconds was applied before initiating the diagnostic scan to ensure optimal contrast opacification during the target aortic phase.

Injector Protocol (Dual-Phase):

Phase 1 (Contrast Bolus): A total volume of 100 mL (adjusted per institutional protocol based on patient weight/BMI) of non-ionic, low-osmolar contrast medium (350 mgI/mL) was injected at a rate of 4.0 mL/s. Injection ceased automatically upon scan trigger.

Phase 2 (Saline Flush): Immediately following, 32 mL of 0.9% physiological saline was injected at the same rate (4.0 mL/s) to flush residual contrast from the

peripheral and central venous system.

2. For CT pulmonary angiography (CTPA):

Trigger Location and Threshold: Patients were stratified into two groups based on history and non-contrast CT images: those with heart failure/pulmonary hypertension, and those without such complaints. For patients with heart failure/pulmonary hypertension, the ROI was placed in the main pulmonary artery. For patients without these complaints, the ROI was placed in the superior vena cava (SVC) at the same axial level as the main pulmonary artery. The automatic triggering threshold was set at 60 HU for both groups.

Trigger-to-Scan Delay: A standard delay of 4 seconds was applied after reaching the threshold before initiating the diagnostic scan.

Injector Protocol (Dual-Phase):

Phase 1 (Contrast Bolus): A total volume of 50 mL (adjusted per institutional protocol based on patient weight/BMI) of the same contrast medium was injected at a rate of 4.5 mL/s.

Phase 2 (Saline Flush): This was followed by 30 mL of 0.9% physiological saline injected at the same rate (4.5 mL/s).

All injections were performed using the same Ulrich Missouri XD2001 CT injector system. This injector was calibrated monthly according to both manufacturer specifications and hospital quality control guidelines. The use of a single injector

and standardized tubing for all study participants was intended to eliminate device-specific variability.

Supplementary Results

Table S1 Subgroup analysis of device effects on SVC artifacts reduction restricted to PCTA without embolism

Characteristics	Novel protocol group (N = 20)	Conventional group (N = 20)	P value	$P_{FDR\ corr}$
Sex				
Male	14	13	0.736	0.968
Female	6	7		
Age, years	63.55 ± 12.65	63.40 ± 10.72	0.968	0.968
Height (cm)	167.95 ± 9.05	164.50 ± 8.94	0.233	0.741
Weight (kg)	64.10 ± 6.92	63.6 ± 9.05	0.845	0.968
BMI (kg/m2)	22.81 ± 2.75	23.48 ± 2.55	0.432	0.741
Pulmonary_Trunk_HU	380.37 ± 47.18	365.80 ± 47.84	0.338	0.741
Left_Pulmonary_Artery_HU	348.09 ± 43.70	346.47 ± 39.75	0.903	0.968
Right_Pulmonary_Artery_HU	342.10 ± 42.34	342.71 ± 45.25	0.965	0.968
Superior_vena_cava_HU	177.15 ± 42.05	603.31 ± 384.20	<0.001	<0.001
Radiation_Dose_CTDIVol	3.19 ± 0.13	3.24 ± 0.14	0.261	0.741
Radiation_Dose_DLP	239.97 ± 14.98	243.98 ± 16.29	0.423	0.741
Contrast_agent_usage	24.35 ± 2.21	38.00 ± 2.51	<0.001	<0.001
Image quality score				
>3	20	8	<0.001	<0.001
≤3	0	12		
Artifacts in superior vena cava				
<2	19	5	<0.001	<0.001
≥2	1	15		

Note: SVC, superior vena cava; PCTA, pulmonary CTA; DLP, Dose-Length Product; CTDIVol, Volume Computed Tomography Dose Index. FDR correction, Benjamini-Hochberg (BH) method.

Table S2 Subgroup analysis of device effects on SVC artifacts reduction restricted to PCTA with embolism

Characteristics	Novel protocol group (N = 12)	Conventional group (N = 12)	P value	$P_{FDR\ corr}$
Sex				
Male	9	8	0.653	0.831
Female	3	4		
Age, years	61.33 ± 11.73	63.33 ± 8.40	0.636	0.831
Height (cm)	169.83 ± 7.64	167.50 ± 7.69	0,464	0.831
Weight (kg)	70.50 ± 10.30	67.67 ± 9.57	0.492	0.831
BMI (kg/m2)	24.34 ± 2.30	24.04 ± 2.26	0.75	0.875
Pulmonary_Trunk_HU	382.97 ± 49.24	372.41 ± 44.84	0.588	0.831
Left_Pulmonary_Artery_HU	351.62 ± 48.68	350.07 ± 46.43	0.937	0.965
Right_Pulmonary_Artery_HU	346.83 ± 46.50	345.92 ± 54.33	0.965	0.965
Superior_vena_cava_HU	178.67 ± 29.51	615.58 ± 269.26	<0.001	<0.001
Radiation_Dose_CTDIVol	3.18 ± 0.12	3.26 ± 0.14	0.139	0.366
Radiation_Dose_DLP	235.67 ± 12.79	244.50 ± 16.52	0.157	0.366
Contrast_agent_usage	24.33 ± 1.56	38.33 ± 2.46	<0.001	<0.001
Image quality score				
>3	12	2	<0.001	<0.001
⩽3	0	10		
Artifacts in superior vena cava				
<2	12	0	<0.001	<0.001
⩾2	0	12		

Note: SVC, superior vena cava; PCTA, pulmonary CTA; DLP, Dose-Length Product; CTDIVol, Volume Computed Tomography Dose Index. FDR correction, Benjamini-Hochberg (BH) method.

Table S3 Subgroup analysis of device effects on SVC artifacts reduction restricted to routine aortic CTA

Characteristics	Novel protocol group (N = 15)	Conventional group (N = 15)	P value	$P_{FDR\ corr}$
Sex				
Male	12	12	1	
Female	3	3		--
Age, years	52.60 ± 15.20	50.33 ± 16.23	0.696	0.809
Height (cm)	166.93 ± 5.84	170.00 ± 10.15	0.319	0.547
Weight (kg)	61.47 ± 8.76	67.00 ± 12.87	0.18	0.54
BMI (kg/m2)	21.95 ± 1.98	23.19 ± 4.19	0.314	0.547
Ascending_Aorta_HU	443.71 ± 40.01	458.85 ± 25.93	0.229	0.547
Descending Aorta at the level of T12 HU	449.64 ± 28.01	442.42 ± 30.31	0.504	0.697
Left_External_Iliac_Artery_HU	430.47 ± 36.56	438.53 ± 31.48	0.523	0.697
Right_External_Iliac_Artery_HU	434.78 ± 42.63	439.49 ± 34.59	0.742	0.809
Superior_vena_cava_HU	159.57 ± 41.00	333.05 ± 293.39	0.039	0.234
Radiation_Dose_CTDIVol	7.71 ± 0.92	8.13 ± 0.70	0.166	0.54
Radiation_Dose_DLP	567.09 ± 79.08	564.40 ± 62.56	0.918	0.918
Contrast_agent_usage	59.40 ± 3.11	74.00 ± 5.07	<0.001	<0.001
Image quality score				
>3	14	5	0.001	0.0047
≤3	1	10		
Artifacts in superior vena cava				
<2	14	5	0.001	0.0047
≥2	1	10		

Note: SVC, superior vena cava; DLP, Dose-Length Product; CTDIVol, Volume Computed Tomography Dose Index. FDR correction, Benjamini-Hochberg (BH) method.

Table S4 Subgroup analysis of device effects on SVC artifacts reduction restricted to post-stent aortic CTA

Characteristics	Novel protocol group (N = 25)	Conventional group (N = 25)	P value	$P_{FDR\ corr}$
Sex				
Male	21	20	0.713	0.713
Female	4	5		
Age, years	60.16 ± 10.00	61.64 ± 8.84	0.582	0.698
Height (cm)	167.08 ± 7.95	166.08 ± 8.86	0.676	0.713
Weight (kg)	66.94 ± 12.20	63.60 ± 7.79	0.254	0.435
BMI (kg/m2)	24.13 ± 5.00	23.05 ± 2.12	0.323	0.485
Ascending_Aorta_HU	448.24 ± 28.67	464.16 ± 42.36	0.126	0.252
Descending Aorta at the level of T12 HU	439.11 ± 28.52	468. 33 ± 36.73	0.003	0.018
Left_External_Iliac_Artery_HU	428.07 ± 48.21	454.57 ± 37.55	0.035	0.105
Right_External_Iliac_Artery_HU	441.56 ± 44.63	449.81 ± 47.47	0.53	0.698
Superior_vena_cava_HU	162.33 ± 25.73	221.06 ± 69.30	0.001	0.012
Radiation_Dose_CTDIVol	8.19 ± 0.6	8.51 ± 0.40	0.028	0.105
Radiation_Dose_DLP	607.14 ± 69.21	573.52 ± 55.39	0.064	0.154
Contrast_agent_usage	59.60 ± 1.38	72.60 ± 4.81	<0.001	<0.001
Image quality score				
>3	24	12	<0.001	<0.001
≤3	1	13		
Artifacts in superior vena cava				
<2	24	12	<0.001	<0.001
≥2	1	13		

Note: SVC, superior vena cava; DLP, Dose-Length Product; CTDIVol, Volume Computed Tomography Dose Index. FDR correction, Benjamini-Hochberg (BH) method.

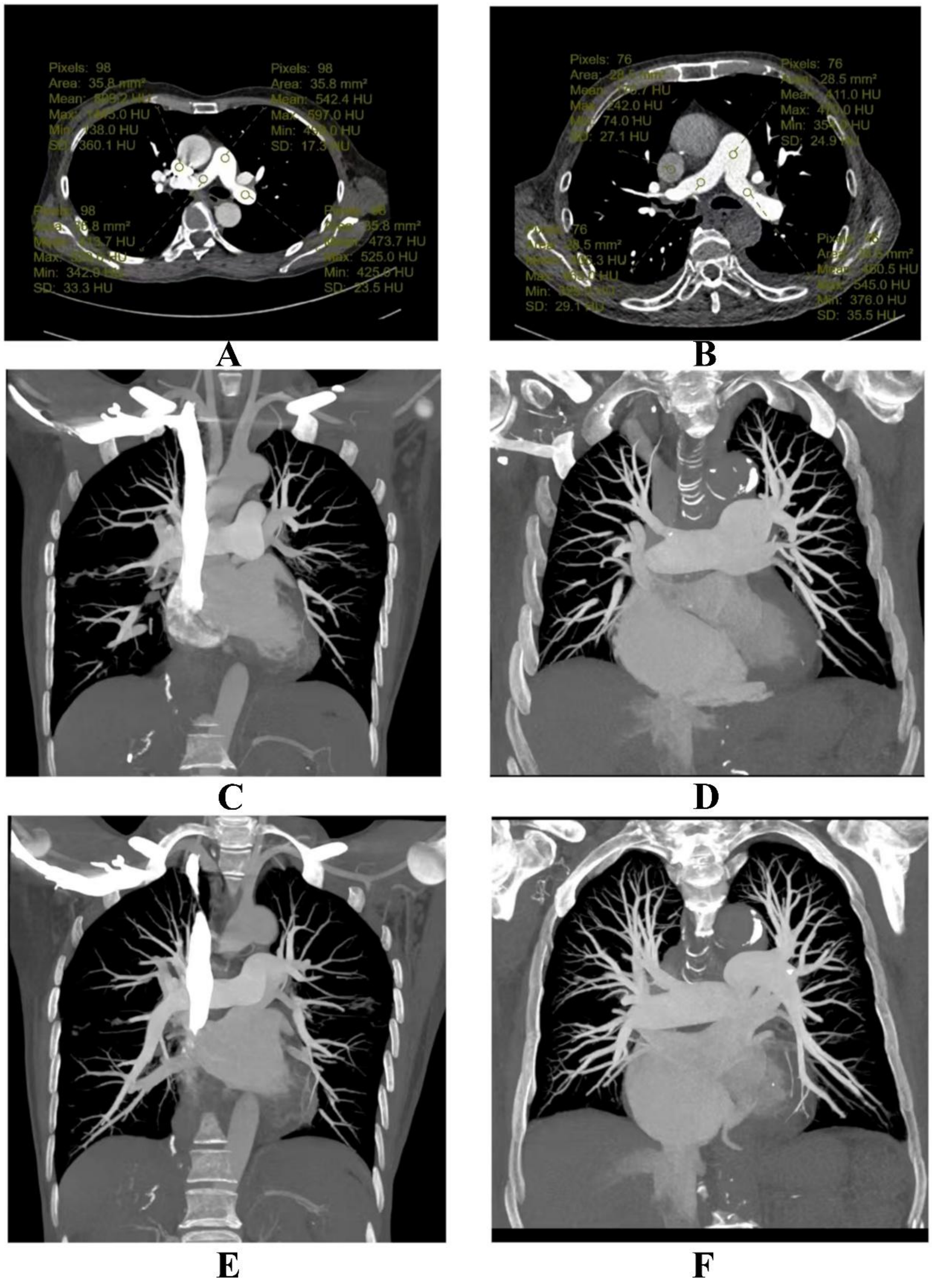

Figure S1. Example of device effects on SVC artifacts reduction restricted to computed tomography pulmonary angiography (CTPA) without embolism. Axial (A) and coronal view (C, E, Maximum Intensity Projection, MIP) of a patient in

conventional group demonstrate well-developed contrast enhancement. The images are sharp and clear; however, a substantial amount of contrast agent within the superior vena cava interferes with the visualization of the right pulmonary artery. By contrast, Axial (B) and coronal view (D, F, Maximum Intensity Projection, MIP) of a patient in the novel protocol group exhibit excellent image quality with distinct clarity with high-quality imaging devoid of artefacts from the superior vena cava.

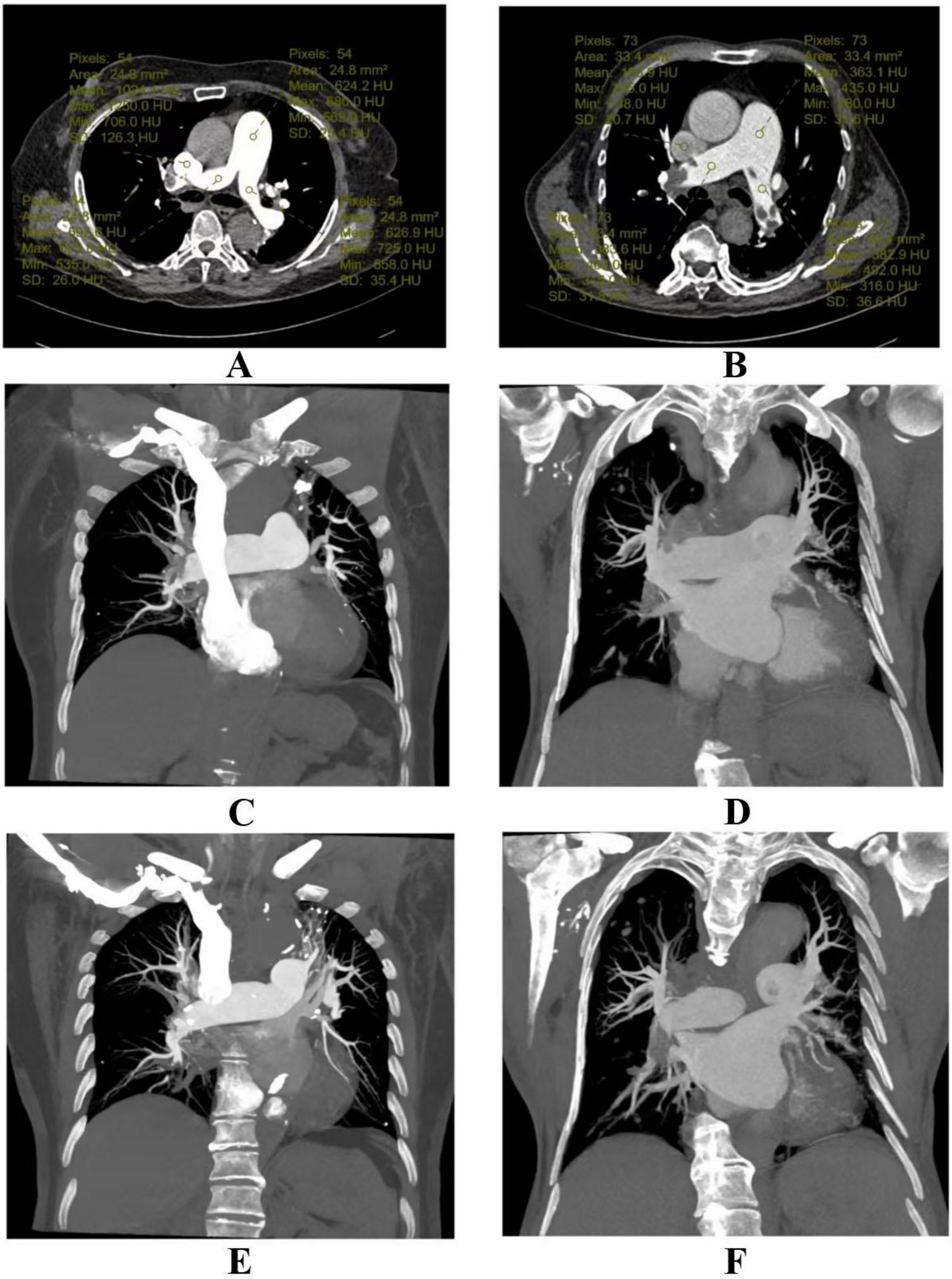

Figure S2. Figure S1. Example of device effects on SVC artifacts reduction restricted to computed tomography pulmonary angiography (CTPA) with embolism. Axial (A) and coronal view (C, E, Maximum Intensity Projection, MIP) of a patient in conventional group demonstrate well-developed contrast

enhancement. The images are sharp and clear; however, a substantial amount of contrast agent within the superior vena cava interferes with the visualization of the embolism in the right pulmonary artery. By contrast, Axial (B) and coronal view (D, F, Maximum Intensity Projection, MIP) of a patient in the novel protocol group exhibit excellent image quality with distinct clarity with high-quality imaging devoid of artefacts from the superior vena cava.

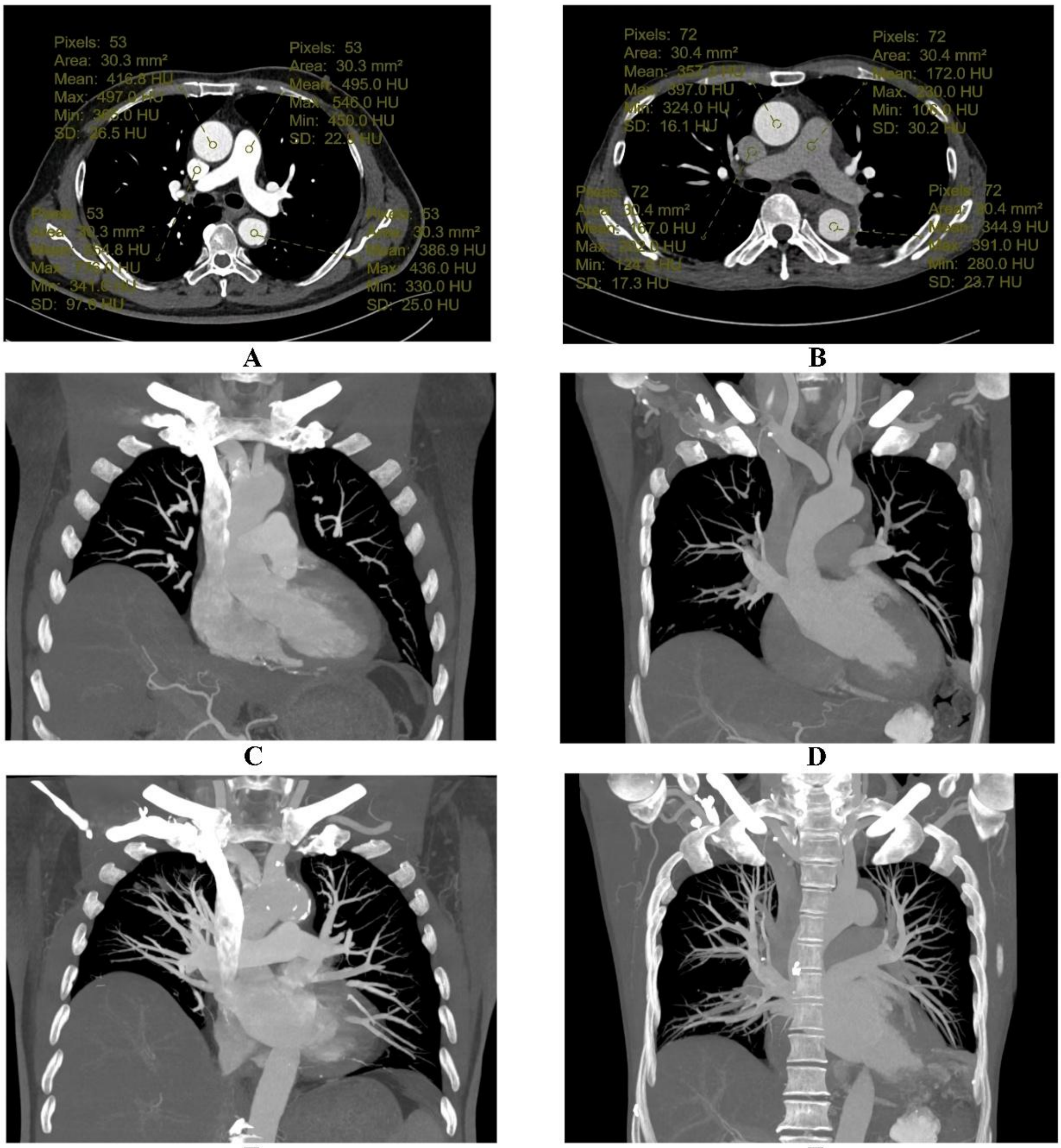

Figure S3. Example of device effects on SVC artifacts reduction restricted to routine aortic CTA. Axial (A) and coronal view (C, E, Maximum Intensity Projection, MIP) of a patient in conventional group demonstrate well-developed contrast enhancement. The images are sharp and clear; however, a substantial amount of contrast agent within the superior vena cava interferes with the aorta. By contrast, Axial (B) and coronal view (D, F, Maximum Intensity Projection, MIP) of a patient in the novel protocol group exhibit excellent image quality with distinct clarity with high-quality imaging devoid of artefacts from the superior vena cava.

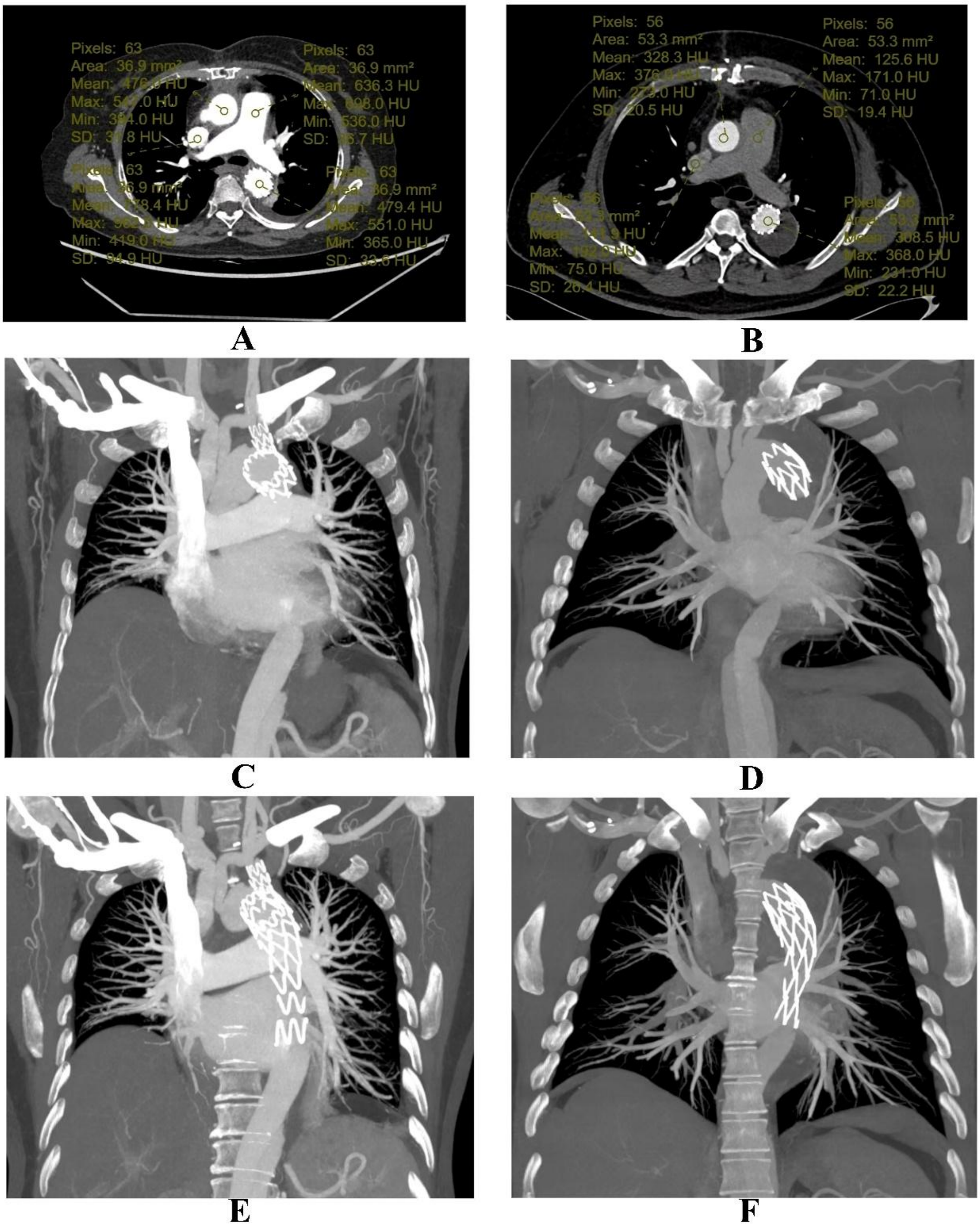

Figure S4. Example of device effects on SVC artifacts reduction restricted to post-stent aortic CTA. Axial (A) and coronal view (C, E, Maximum Intensity Projection, MIP) of a patient in conventional group demonstrate well-developed contrast enhancement. The images are sharp and clear; however, a substantial amount of contrast agent within the superior vena cava interferes with the aorta. By contrast,

Axial (B) and coronal view (D, F, Maximum Intensity Projection, MIP) of a patient in the novel protocol group exhibit excellent image quality with distinct clarity with high-quality imaging devoid of artefacts from the superior vena cava.
